# Effects of Roasting Conditions on the Quality of Sesame Oil: Sensory Profiles, Volatile Components, Fatty Acids and Oxidative Stability

**DOI:** 10.3390/foods15010146

**Published:** 2026-01-02

**Authors:** Mengke Zheng, Yan Chen, Peiwen Yang, Yinan Yang, Guihong Qi, Peng Li, Wuduo Zhao, Shihao Sun, Donghao Zhang

**Affiliations:** 1 Flavor Science Research Center, College of Chemistry, Zhengzhou University, Zhengzhou 450001, China; 2 Beijing Life Science Academy, Beijing 102209, China

**Keywords:** roasting conditions, sesame oil, sensory profiles, fatty acids, oxidative stability

## Abstract

Sesame oil is one of the most popular sesame products for consumers. Roasting is a commonly employed heat treatment method in sesame oil processing. This work aims to investigate the effects of roasting temperature and time on sensory profiles, volatile components, fatty acid composition, and oxidative stability of the oil. Quantitative descriptive sensory analysis was employed to reveal changes in aroma characteristics of sesame oils from different roasting conditions. Volatile compounds of the oils were analyzed via headspace solid-phase microextraction/gas chromatography–mass spectrometry (HS-SPME/GC-MS), identifying 56 components, including 30 key aroma-active compounds (odor activity value, OAV ≥ 1) across 18 samples. Principal component analysis (PCA) was performed to explore the effect of roasting conditions on volatiles of sesame oils. The oxidative stability of the oils was also determined by RapidOxy reactor. The results demonstrated that the effect of roasting time on the flavor of sesame oil was greater than that of temperature. Moreover, the effect of roasting conditions (temperature/time) on the fatty acid profile of sesame oil was not significant. This provided some theoretical foundation and data support for improving the processing technology of sesame oil and controlling its flavor quality.

## 1. Introduction

Sesame (*Sesamum indicum* L.) is planted worldwide as an oil seed crop in tropical and sub-tropical areas [1,2]. Sesame seeds are rich in oil, fatty acids, protein, and bioactive components such as lignans and tocopherols [3]. Sesame oil is one of the most popular sesame products for consumers, which is obtained from sesame seeds through various processing techniques [4]. It contains numerous bioactive components such as sesamin, sesamol and sesamolin, which have potential benefits for anti-aging, hypercholesterolemia, hypertension, and cancer treatment [5,6]. The high nutritional value, unique flavor and superior oxidative stability of sesame oil are the primary reasons for its consumer appeal [7].

The production technology of sesame oil mainly includes mechanical expression, solvent extraction and hot water flotation [8,9]. The initial steps of these techniques are generally similar, including washing, drying, crushing and roasting of sesame seeds. Among these, roasting is the most critical step affecting the quality of sesame oil [10]. Roasting conditions not only affect the extraction rate of sesame oil, but also affect the nutritional value, oxidative stability and sensory acceptance [11,12]. The main reason is that the roasting process would lead to many complex reactions, including the Maillard reaction, sugar degradation, protein denaturation and lipid oxidation, etc. [13,14]. The complex reaction products generated during roasting could enhance the characteristic flavor of sesame oil and improve its oxidative stability [15]. Some studies have shown that increasing roasting temperature was beneficial to the formation of characteristic flavor components of sesame oil, such as pyrazines and furans [16,17]. However, inappropriate roasting conditions will cause adverse effects; some potentially toxic compounds would be formed in this process, e.g., 5-hydroxymethyl-furfural (HMF), acrylamide, and furan, which may have a potential impact on human health [18].

While temperature and time are undeniably crucial factors in the roasting process, previous studies have often focused on the impact of a single parameter on specific quality attributes, such as flavor or stability, over a limited range of conditions [19,20]. It is worth noting that no relevant study has simultaneously included the roasting temperature and time into the scope of the study. It is of great significance to clarify the key aroma components that determine the flavor quality or difference in sesame at different roasting levels, because it can guide sesame oil producers to better control the consistency of product quality. In addition, it is important to optimize the sesame roasting process. This is because even under the same roasting degree, different roasting profiles (time-temperature) will lead to very different volatile and non-volatile compounds in sesame [21]. When the key aroma compounds that determine or characterize the roasting degree of sesame are identified, it will provide more scientific guidance for the improvement of roasting process and roasting conditions. Therefore, a more detailed description of different roasting degrees and the exploration of the differences in key aroma compounds at different stages will lay a solid material foundation for clarifying the quality differences in sesame oil flavor under different roasting degrees. In addition, the understanding of the compounds that lead to the difference in sensory properties of sesame oil under different roasting degrees is very limited, so it is difficult to really reveal the material basis of flavor components that determine consumer acceptance and consumer preference [22]. This is not conducive to sesame oil producers to improve the quality of sesame oil and further develop products that cater to consumers’ eating preferences. Therefore, it is necessary to comprehensively analyze sesame oil with different roasting degrees. Furthermore, oils are prone to oxidative deterioration during storage. This makes obtaining superior flavor characteristics while ensuring storage quality an important approach to achieving high-quality sesame oil [9]. However, there has been no research exploring the dual impact of different roasting levels on the flavor and oxidative stability of sesame oil, making it difficult for manufacturers to achieve a high-quality balance between flavor quality and storage quality.

This study therefore systematically examined the combined effects of roasting time (10–30 min) and temperature (160–210 °C) on the quality of sesame oil. Initially, sensory evaluation was conducted to explore flavor variations. Volatile compounds were qualitatively and quantitatively analyzed using headspace solid-phase microextraction/gas chromatography–mass spectrometry (HS-SPME/GC-MS), with key odor-active compounds identified through odor activity value (OAV) analysis. Subsequently, multivariate statistical analysis was employed to investigate the relationships between roasting treatments and volatile compounds. Additionally, fatty acid composition and oxidative stability were systematically characterized.

## 2. Materials and Methods

### 2.1. Chemicals and Materials

Pre-packaged white sesame seeds were obtained from a local supermarket in Zhengzhou, Henan Province, China, and stored at 4 °C in a refrigerator until further use. 2-Methyl-3-heptanone and a mixture of n-alkanes (C7–C30) were purchased from Merck (Darmstadt, Germany). Isooctane, potassium hydroxide (KOH), methanol, acetic acid, and anhydrous sodium hydrogen sulfate were supplied by Aladdin Biochemical Technology Co., Ltd. (Shanghai, China). Potassium iodide, chloroform, sodium thiosulfate, and starch indicator were purchased from Macklin Biochemical Co., Ltd. (Shanghai, China).

### 2.2. Sesame Oil Samples Preparation

Based on the work of Ji et al. [10], this study selected temperature and time parameters that more closely reflect actual production conditions. Specifically, sesame seeds (500 g) were roasted by a preheated Galanz conventional oven (Guangdong, China) under different temperatures (160 °C, 170 °C, 180 °C, 190 °C, 200 °C, 210 °C) and for different times (10, 20 and 30 min), encompassing all 18 possible temperature-time combinations. Then, sesame seeds were crushed by a Joyoung blender (Shandong, China). Sesame seed powder (100 g) was shaken with n-hexane (500 mL) for 3 h and extracted in the dark at constant temperature for 12 h, the supernatant was transferred for subsequent use. The excess solvent in the supernatant was removed using a rotary evaporator (BUCHI Labortechnik AG R210, Shanghai, China), and the sample was finally centrifuged at 12,000 rpm for 20 min at 4 °C using a benchtop high-speed refrigerated centrifuge (Sigma 2-16KL, Osterode am Harz, Germany). The supernatant liquid was sesame oil, and it was stored in brown bottles sealed in a refrigerator at 4 °C for subsequent use.

### 2.3. Sensory Evaluation

Sensory analysis of sesame oils obtained under different roasting conditions was conducted using the Quantitative Descriptive Analysis (QDA) method. Sensory evaluators were recruited from the Flavor Research Center at the College of Chemistry, Zhengzhou University. After screening, a standardized sensory evaluation panel comprising 10 assessors (5 males and 5 females, aged 22–30 years) was established. Written informed consent was obtained from all participants, and ethical protocols were strictly followed to protect volunteers’ rights and privacy. Through panel discussions and literature review, six aroma descriptors were identified: nutty, sweet, roasted, burnt, green, and pungent. Intensity scales were established through comprehensive calibration sessions using representative sesame oil samples spanning the intensity range for each attribute. Unroasted sesame oil was included as a control sample in all evaluations to provide a sensory baseline for comparison. All samples were evaluated in a standardized sensory analysis room (23 ± 1 °C), where samples were randomly coded and sequentially assessed by panelists using the QDA protocol, with a mandatory 1 min interval between every two samples to minimize olfactory fatigue-induced variability.

### 2.4. HS-SPME/GC-MS Analysis of Sesame Oil

The HS-SPME method of volatile components from sesame oil was our previous method and some modifications have been made [23]. A 50/30 μm DVB/CAR/PDMS SPME fiber (Supelco, Inc., Bellefonte, PA, USA) was pretreated at 270 °C for 30 min before each experiment. Sesame oil (1 g) and 2-methyl-3-heptanone (1 µL, 0.816 µg/µL) were immediately transferred to a 4 mL headspace sample vial, and the cap was quickly tightened. The headspace sample vial was pre-equilibrated in a 60 °C thermostatic water bath for 20 min. The aged DVB/CAR/PDMS fiber was then driven into the vial to extract the volatile compounds at 60 °C for 60 min.

The volatile compounds were analyzed by an Agilent 7890B gas chromatograph coupled with a 5977B series mass spectrometer (Santa Clara, CA, USA). The DB-WAXETR column system (60 m × 250 μm × 0.25 μm) was adopted to separate volatile compounds. The volatile components were desorbed at the injector at 250 °C for 5 min in splitless mode. The flow rate of helium as carrier gas is 1.0 mL/min. The initial oven temperature was 40 °C, then raised to 170 °C at 2.5 °C/min and held for 3 min. Finally, ramped up to 240 °C at 10 °C/min for 10 min. The MS parameters were set as follows: ion source temperature, 230 °C; ionization energy, 70 eV; and scan mass range, 30–350 *m*/*z*.

### 2.5. Identification, Quantification, and OAVs Calculation of the Volatile Compounds

The qualitative analysis of volatile compounds was realized by comparing the mass spectrum information of volatile compounds with the NIST 17.0 database. The retention index of each compound was calculated using the following equation and further characterized by comparison with the literature.

$$RI=100n+100\times \frac{t_{Ru}-t_{Rn}}{t_{R(n+1)}-t_{Rn}}$$

where *n* is the number of carbon atoms in each n-alkane, *t_R_*_u_ is the retention time of the volatile compound, *t_R_*_n_ is the retention time of n-alkanes with carbon number *n*, and *t_R_*_(n+1)_ is the retention time of n-alkanes with carbon number *n* + 1.

The relative concentration of volatile compounds was determined by using a semi-quantitative method with 2-methyl-3-heptanone as an internal standard [22]. The OAVs were calculated by dividing the relative concentration of the volatile compounds by their odor thresholds.

### 2.6. Fatty Acid Composition Analysis

Fatty acids were esterified as fatty acid methyl esters (FAMEs) for further analysis. FAMEs were prepared following a previously reported method with appropriate modifications [24]. A mixture of 60 mg of sesame oil and 4 mL of isooctane was vortexed for 1 min. Then, 200 μL of methanolic KOH (2 mol/L) was added, and the mixture was vortexed for an additional 1 min. Anhydrous sodium bisulfate was added to the mixture, which was left to stand for 20 min, and the upper layer was collected for analysis.

FAMEs were determined by an Agilent 7890B gas chromatograph coupled with a 5977B series mass spectrometer (Santa Clara, CA, USA) and DB-WAXETR column (60 m × 250 μm × 0.25 μm). The injection port temperature was 250 °C in splitless mode. The flow rate of helium as carrier gas is 1.0 mL/min. The initial oven temperature was 120 °C for 3 min, then raised to 200 °C at 4 °C/min and held for 10 min. Finally, ramped up to 240 °C at 2 °C/min for 10 min. The MS parameters were set as follows: ion source temperature, 230 °C; ionization energy, 70 eV; and scan mass range, 30–550 *m*/*z*.

### 2.7. Peroxide Value

Peroxide value (POV) is a primary measure of oxidative deterioration in edible oils and fats. POV was determined according to ISO 3960:2017 [25,26]. Briefly, 5.0 g of the sesame oil sample was dissolved in an acetic acid–chloroform mixture (3:2, *v*/*v*). Subsequently, 1 mL of saturated potassium iodide solution was added, and the mixture was vigorously shaken and kept in the dark for 1 min. The liberated iodine was then titrated with 0.01 mol/L sodium thiosulfate solution using starch as the indicator. The results were expressed as mmol peroxide/kg oil.

### 2.8. Determination of Oxidative Stability

The oxidative stability of sesame oil, a crucial indicator of its resistance to oxidative degradation and shelf-life, was determined on a RapidOxy reactor (Anton Paar, Blankenfelde-Mahlow, Germany) according to the method of Rodríguez et al., with appropriate modification [27]. This accelerated oxidation test exposes the sample to elevated temperature and high-pressure oxygen. Briefly, a 3 g sample was heated in a sealed chamber at 150 °C under an initial oxygen pressure of 700 kPa. As oxidation proceeds, oxygen is consumed, leading to a measurable pressure drop. The oxidation induction time (OIT), defined as the time required to achieve a 10% pressure drop, was recorded. The OIT represents the period during which the oil’s inherent antioxidants inhibit rapid oxidation. Once depleted, oxidation accelerates autocatalytically. Therefore, a longer induction time corresponds to greater oxidative stability and stronger antioxidant protection.

### 2.9. Statistical Analysis

SPSS 26.0 (IBM Corp., Armonk, NY, USA) was used to perform a one-way ANOVA, with statistical significance set at *p* < 0.05. Principal Component Analysis (PCA) was conducted using SIMCA 14.1 (Umetrics, Umeå, Sweden), and heat maps were generated using TBtools (Toolbox for Biologists; version 1.082, Guangzhou, China). Data obtained from the experiment were analyzed using Origin 2018 (Origin Lab Corporation, Northampton, MA, USA). Each experiment was repeated three times to ensure reliability.

## 3. Results and Discussion

### 3.1. Sensory Characteristics of Sesame Oil Under Different Roasting Conditions

**Figure 1:**
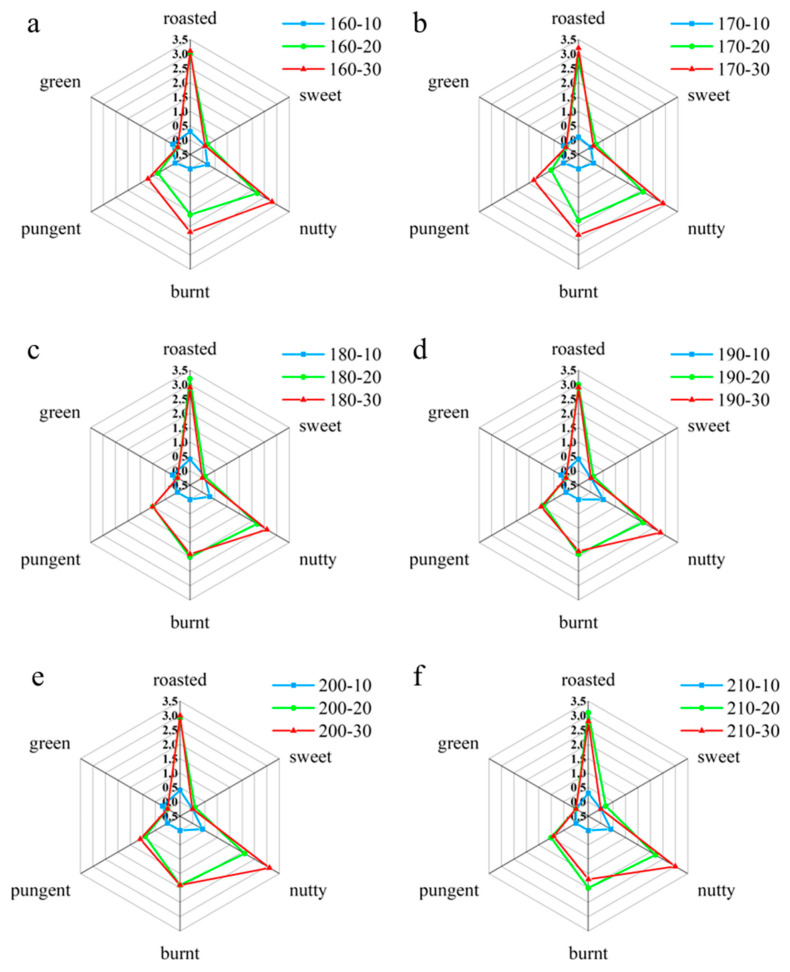
Sensory characteristics profile analysis of sesame oil aroma under different roasting conditions. (**a**) Roasted at 160 °C; (**b**) Roasted at 170 °C; (**c**) Roasted at 180 °C; (**d**) Roasted at 190 °C; (**e**) Roasted at 200 °C; (**f**) Roasted at 210 °C.

According to the QDA results, the aroma profiles of sesame oil under different roasting conditions were plotted as shown in Figure 1. These profiles revealed significant differences, with the overall effect of roasting time being more pronounced in shaping the sensory characteristics of the oil.

Specifically, when the roasting time was 10 min and the temperature was below 170 °C, the flavor characteristics of the sesame oil were weak, exhibiting only faint roasted, nutty, and green aromas. As the roasting time was extended to 20 and 30 min, the intensities of the nutty and roasted aromas gradually increased. This enhancement of roasted and nutty notes is a well-documented outcome of Maillard reactions and sugar caramelization during roasting, consistent with observations in other roasted oilseeds [28]. Under the conditions of 20 and 30 min of roasting at 160–210 °C, the flavor profiles of the 12 sesame oil samples became more prominent, primarily characterized by roasted, nutty, and burnt aromas, accompanied by a certain sweet and green aroma. The analysis showed that the roasting time mainly affected the intensities of the roasted, nutty and burnt aromas, while it had less impact on the sweet and green aromas. Therefore, appropriately extending the roasting time helped to enhance these specific flavors. However, as roasting time and temperature increased, some pungent odors, possibly due to the formation of certain phenolic compounds, also became more intense. Notably, when the roasting time is extended to 30 min, the sesame oil develops a pronounced burnt odor, which compromises consumers’ sensory acceptance. Our sensory evaluation indicated that for a 20 min roasting duration, maintaining the temperature below 170 °C was crucial for preventing the emergence of off-flavors and preserving an optimal aroma balance.

### 3.2. HS-SPME/GC-MS Analysis of Sesame Oil Samples

**Figure 2:**
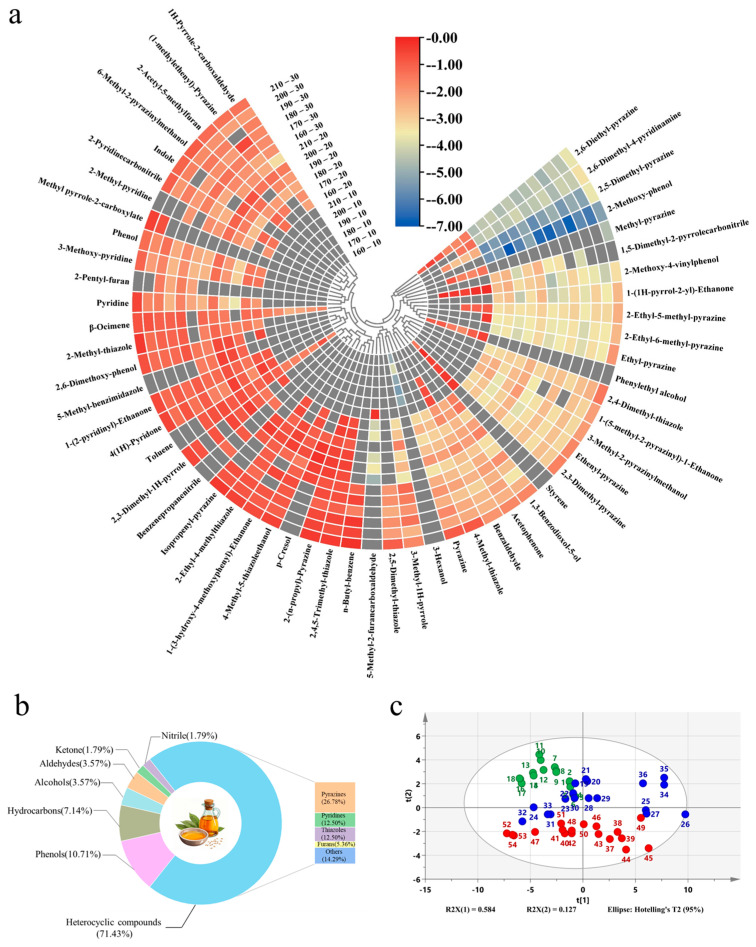
Cluster heatmap of volatile compounds in sesame oil samples (**a**). Categories and percentages of volatile compounds in sesame oil (**b**). PCA score plot of volatile compounds in sesame oil samples obtained under different roasting conditions (**c**). Note: 1–3: Roasted at 160 °C for 10 min; 4–6: Roasted at 170 °C for 10 min; 7–9: Roasted at 180 °C for 10 min; 10–12: Roasted at 190 °C for 10 min; 13–15: Roasted at 200 °C for 10 min; 16–18: Roasted at 210 °C for 10 min; 19–21: Roasted at 160 °C for 20 min; 22–24: Roasted at 170 °C for 20 min; 25–27: Roasted at 180 °C for 20 min; 28–30: Roasted at 190 °C for 20 min; 31–33: Roasted at 200 °C for 20 min; 34–36: Roasted at 210 °C for 20 min; 37–39: Roasted at 160 °C for 30 min; 40–42: Roasted at 170 °C for 30 min; 43–45: Roasted at 180 °C for 30 min; 46–48: Roasted at 190 °C for 30 min; 49–51: Roasted at 200 °C for 30 min; 52–54: Roasted at 210 °C for 30 min (**c**).

Flavor is one of the fundamental characteristics of edible oils, which is mainly influenced by volatile components [29]. In this study, HS-SPME/GC-MS was employed to analyze volatile compounds in sesame oil produced under various roasting conditions. As shown in Table A1 and Figure 2b, a total of 56 volatile compounds were detected across 18 sesame oil samples, including 40 heterocyclic compounds, 6 phenols, 4 hydrocarbons, 2 alcohols, 2 aldehydes, 1 ketone, and 1 nitrile. PCA of these volatile components (Figure 2c) revealed that samples grouped distinctly by roasting time, suggesting that roasting duration has a more pronounced influence on the volatile profile than temperature alone.

When roasting time was extended to 20 and 30 min, heterocyclic compounds became the predominant volatile class, with concentrations ranging from 102.77 to 316.94 μg/g (Figure 2a). These mainly include pyrazines, pyridines, thiazoles and furans. With the change in roasting temperature and time, the content of heterocyclic compounds reached the highest at 210 °C for 20 min, which was 316.94 μg/g. Pyrazines were the most abundant heterocyclic compounds in roasted sesame oil, which contributed to the roasty, nutty, and popcorn-like aromas [30,31], and their formation is primarily attributed to the Maillard reaction under high temperature conditions, explaining their elevated levels in prolonged, high temperature roasting [32]. This aligns with the established trend in hot-air roasting studies where extended thermal treatment promotes Maillard-derived pyrazine formation [25]. Additionally, with the increase in roasting temperature and time, different degrees of O-heterocyclic compounds were also found in sesame oil samples. Such as 2-pentyl-furan and 5-methyl-2-furancarboxaldehyde, which likely contribute sweet notes to the oil [33].

Phenols were detected in all samples except those roasted at 160 °C for 10 min, with concentrations ranging from 0.06 to 150.03 μg/g. These compounds impart smoky and sweet notes; however, due to their low sensory thresholds, high concentrations can easily introduce pungent off-odors. Notably, 2-methoxy-phenol (35.03–124.43 μg/g) was identified only in samples roasted for 20 or 30 min, indicating that longer roasting times promote its formation, likely due to the enhanced thermal degradation of lignin [34]. Interestingly, the concentration of 2-methoxy-phenol and 2,6-dimethoxy-phenol was relatively lower at 170 °C for 20 min compared to other conditions, which may account for the less pungent aroma observed under this specific roasting treatment.

Among alcohols, 3-hexanol (14.89–41.63 μg/g) and phenylethyl alcohol (0.37–2.36 μg/g) were detected, and only in samples roasted for 10 min. This suggests these alcohols are transient intermediates formed during the early stages of roasting through lipid oxidation (e.g., 3-hexanol from unsaturated fatty acid degradation) or amino acid breakdown. With prolonged roasting, their high volatility and susceptibility to thermal degradation or secondary reactions likely cause their disappearance, shifting the flavor profile toward more stable compounds like pyrazines or furans. This observation of early-formed lipid-derived volatiles being supplanted by Maillard reaction products during prolonged heating is consistent with observations in other roasted oilseed systems, such as almonds, where lipid-derived compounds dominate the raw aroma but are overshadowed by thermal reaction products after roasting [28]. Minor amounts of ketones, aldehydes, hydrocarbons, and a nitrile were also identified but likely had limited influence on overall aroma due to their low concentrations.

### 3.3. Analysis of the Key Aroma Compounds OAVs in Sesame Oil

**Figure 3:**
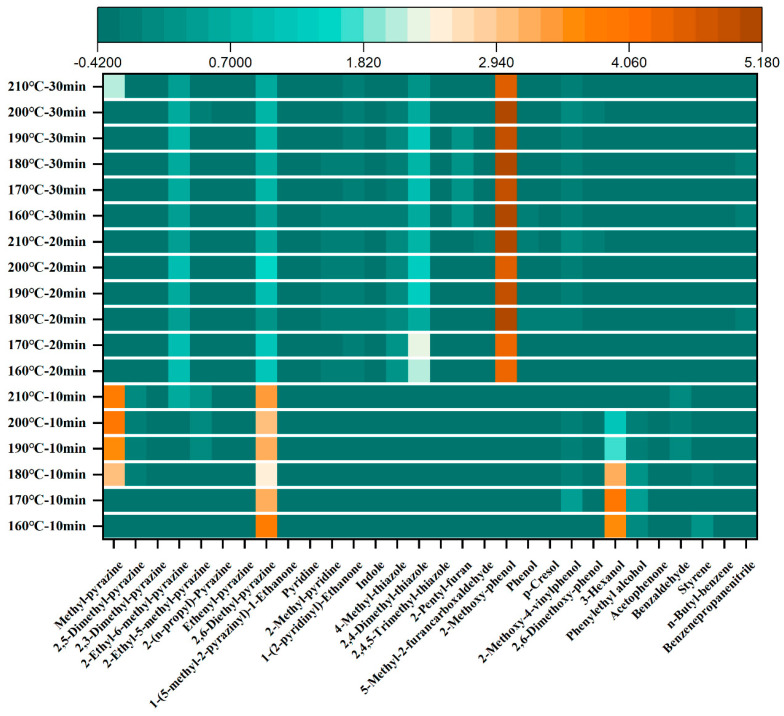
Heatmap analysis of OAVs used to distinguish different roasting conditions (Data standardized by Z-score).

The contribution of volatile compounds to the aroma profile of sesame oil can be determined by calculating the OAVs, and the components with OAVs greater than 1 are usually considered as the key aroma compounds [35]. Based on the qualitative and quantitative results of volatile compounds obtained above, combined with odor thresholds reported in the literature, OAVs were calculated to further investigate the impact of roasting conditions on key aroma-active compounds in sesame oil. As shown in Table A2 and Figure 3, 30 aroma active compounds (OAVs ≥ 1) were identified in 18 samples, including 18 heterocyclic compounds, 5 phenols, 2 alcohols, 1 aldehyde, 1 ketone and 3 other compounds. Samples roasted for 10 min contained fewer odor-active compounds, mainly pyrazines (e.g., 2,6-diethyl-pyrazine) and alcohols (e.g., 3-hexanol), imparting a mild nutty and green aroma to the resulting oil, which is consistent with the sensory evaluation results. In contrast, samples roasted for 20 or 30 min exhibited a greater number and diversity of odor-active compounds, including several phenols in addition to heterocyclics. This observation is consistent with prior studies that identified pyrazines, furans, and phenols as key aroma-active compounds in roasted sesame oil, contributing significantly to its characteristic nutty, roasted, and burnt profiles [30]. 2-Methoxy-phenol exhibited the highest OAVs (701–2489), contributing prominent sweet and smoky aromas [36]. This was followed by 2-ethyl-6-methyl-pyrazine (OAV: 128–367) and 2,4-dimethyl-thiazole (OAV: 91–505), which are associated with roasted potato-like notes [37]. These compounds significantly shape the aroma profile of sesame oil and are highly influenced by roasting conditions. However, it is noteworthy that the concentrations of key phenolic compounds, particularly 2-methoxy-phenol (OAV: 815) and 2-methoxy-4-vinylphenol (OAV: 22), were relatively lower in the sample roasted at 170 °C for 20 min compared to other long-duration roasting conditions. This may account for the less pungent and more balanced aroma profile observed under this specific treatment.

### 3.4. Fatty Acid Composition Profile

**Figure 4:**
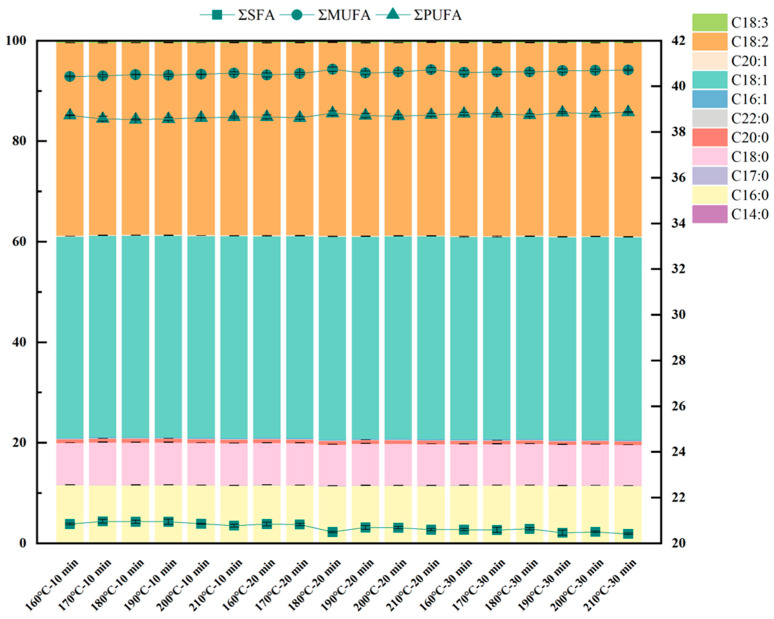
Relative distribution of individual fatty acids and composition of total fatty acid classes (SFAs, UFAs and PUFAs).

The composition of fatty acids reflects, to some extent, the stability and nutritional quality of vegetable oils. In this study, 18 sesame oil samples prepared under different roasting conditions were analyzed using GC-MS, identifying a total of 11 fatty acids (Figure 4), including 6 saturated fatty acids (SFAs), 3 unsaturated fatty acids (UFAs), and 2 polyunsaturated fatty acids (PUFAs). Across all samples, SFAs ranged from 20.41% to 20.96%, UFAs from 40.43% to 40.75%, and PUFAs from 38.55% to 38.87% of the total fatty acids, with no significant differences attributable to roasting parameters (*p* > 0.05). This stability aligns with prior reports on sesame oil [26] and is echoed in studies of other oilseeds, such as peanuts and camellia seeds, where roasting similarly exerts negligible effects on fatty acid profiles [21,38].

Notably, oleic acid (C18:1) and linoleic acid (C18:2) dominated the composition, collectively comprising over 75% of total fatty acids, followed by palmitic acid (C16:0) and stearic acid (C18:0). Roasting for 30 min was found to increase the proportion of linoleic acid in sesame oil samples, which was the highest, accounting for 38.35–38.49%. This predominance of oleic and linoleic acids aligns with the characteristic fatty acid profile reported for sesame oils from diverse geographical origins [24]. While elevated PUFAs content may theoretically compromise oxidative stability, the relationship between PUFAs levels and oxidative stability in sesame oil is not solely deterministic due to the presence of endogenous antioxidants such as tocopherols and lignans, necessitating a comprehensive evaluation of multiple contributing factors [15]. These findings reinforce the potential of optimized roasting to preserve sesame oil’s nutritional integrity alongside its flavor and stability profiles.

### 3.5. Oxidative Stability Analysis

**Figure 5:**
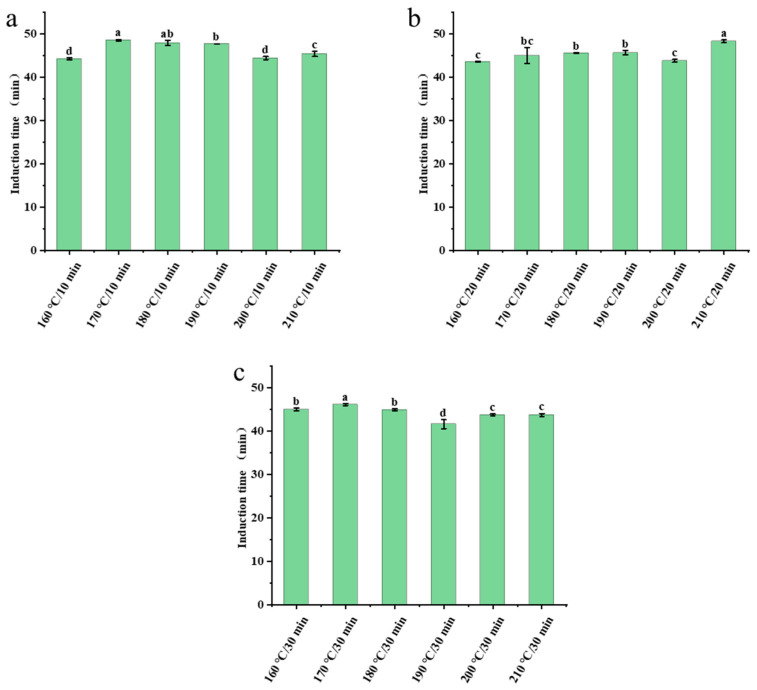
Induction time of sesame oil samples obtained under different roasting conditions. (**a**) Roasted for 10 min; (**b**) Roasted for 20 min; (**c**) Roasted for 30 min. Different letters above bars indicate significant differences (*p* < 0.05).

The oxidative stability and antioxidant activity of the samples were evaluated based on the OIT [39]. In this study, the OIT (41.61–48.56 min) of sesame oil samples was determined under accelerated conditions by the RapidOxy reactor. The trend of oxidative stability varied with roasting temperature and time. When the roasting time was 10 min, the OIT of the sesame oil samples first increased and then decreased with rising roasting temperature, reaching a maximum value of 48.56 min at 170 °C (Figure 5a). A similar trend was observed for the 30 min roasting duration (Figure 5c), with the OIT also peaking at 170 °C (46.13 min) before decreasing. In contrast, at a roasting duration of 20 min, the OIT exhibited a consistent increase with temperature, attaining 48.29 min at 210 °C (Figure 5b).

Moreover, the POVs of all 18 sesame oil samples, which ranged from 0.23 to 2.00 mmol/kg, were well below the limit (7.5 mmol/kg) specified in ISO 3960:2017 [25]. This indicates that the samples possessed very low levels of primary oxidation products at the time of analysis, consistent with the observed relatively high OIT values and reflecting good initial oxidative stability.

The observed trends in OIT can be attributed to the complex chemical transformations occurring during roasting, which are directly linked to the volatile composition detailed in Section 3.2. Specifically, the enhancement of oxidative stability is largely due to the formation of Maillard reaction products and other thermal degradation products with antioxidant properties [40,41]. Our HS-SPME/GC-MS analysis showed that prolonged and high-temperature roasting promoted the formation of various heterocyclic compounds, including pyrazines, pyridines, and furans. Although the antioxidant capacity of these compounds varies considerably depending on their molecular structure, previous studies have demonstrated that certain Maillard-derived heterocyclic compounds, especially substituted furans and pyrroles, can effectively scavenge free radicals and inhibit lipid oxidation [42]. These molecules may therefore contribute to the improved oxidative stability of roasted sesame oil by quenching reactive radicals or interacting with pro-oxidant species, which slows the initiation of lipid oxidation chains. Furthermore, the increase in phenolic compounds, represented by 2-methoxy-phenol under extended roasting times (20 and 30 min), also contributes to the enhanced oxidative stability. Phenolic compounds can function as primary antioxidants by donating hydrogen atoms to quench lipid-derived radicals and interrupt propagation reactions [43]. Thus, the notable effect of roasting on oxidative stability is partly associated with the formation of these antioxidant-active volatile components.

However, this positive effect is counteracted by the thermal degradation of endogenous, heat-sensitive antioxidants naturally present in sesame, including tocopherols and sesamol [44]. The final OIT value, therefore, reflects the combined outcome of the formation of new antioxidant substances, such as heterocyclic Maillard reaction products and phenolic volatiles, and the simultaneous loss of native antioxidants. This balance explains the observed optimum conditions in this study, such as those at 170 °C for 10 min, where the generation of additional antioxidant compounds likely reached a favorable level before the degradation of intrinsic antioxidants became predominant. A similar dual-effect phenomenon has been reported for other plant oils, including chia seed oil and *Pistacia terebinthus* oil [45,46].

## 4. Conclusions

This study investigated the effects of roasting temperatures (160–210 °C) and times (10–30 min) on key quality attributes of sesame oil, with a focus on parameters closely linked to consumer preference and commercial value, including aroma profile, volatile components, and oxidative stability. The results demonstrated that roasting conditions significantly influenced the volatile composition and sensory characteristics, while the fatty acid profile remained largely unchanged. Under short-term roasting (10 min), volatile components were dominated by pyrazines such as 2,6-diethylpyrazine and alcohols, including 3-hexanol, imparting green and nutty aromas. When the roasting time was extended to 20–30 min, heterocyclic compounds and phenols became the predominant components, resulting in an aroma profile characterized by intense roasted and burnt notes. Regarding oxidative stability, the OIT of sesame oil obtained by roasting at 170 °C for 10 min reached a peak value (48.56 min). In conclusion, optimizing roasting conditions (e.g., 170 °C for 10–20 min) effectively enhances the flavor of sesame oil while maintaining its oxidative stability and nutritional value. The findings establish a crucial basis for the systematic optimization of roasting conditions, paving the way for future work that combines the sensory and stability dimensions with a comprehensive evaluation of nutritional and safety aspects to achieve a complete quality profile. Future studies could further employ complementary analytical techniques (e.g., FTIR spectroscopy) to obtain deeper mechanistic insights into the oxidation process. This study provides scientific insights under controlled laboratory conditions, serving as a reference for understanding roasting-induced changes in sesame oil quality. In industrial settings, the specific parameters would still require adjustment according to equipment characteristics.

## Data Availability

The original contributions presented in this study are included in the article. Further inquiries can be directed to the corresponding authors.

## Abbreviations

The following abbreviations are used in this manuscript:

OAVs	odor activity values
FAMEs	fatty acid methyl esters
OIT	oxidation induction time
QDA	quantitative descriptive analysis
SFAs	saturated fatty acids
UFAs	unsaturated fatty acids
PUFAs	polyunsaturated fatty acids
POV	peroxide value

## Appendix A

**Table A1:** Identification of volatile compounds in sesame oil samples.

NO.	Formula	Compounds ^a^	CAS	RI_cal_^b^	RI_ref_^c^
1	C_4_H_4_N_2_	Pyrazine	290-37-9	1213	1204
2	C_5_H_6_N_2_	Methyl-pyrazine	109-08-0	1267	1259
3	C_6_H_8_N_2_	2,5-Dimethyl-pyrazine	123-32-0	1323	1311
4	C_6_H_8_N_2_	Ethyl-pyrazine	13925-00-3	1336	1333
5	C_6_H_8_N_2_	2,3-Dimethyl-pyrazine	5910-89-4	1347	1337
6	C_7_H_10_N_2_	2-Ethyl-6-methyl-pyrazine	13925-03-6	1386	1367
7	C_7_H_10_N_2_	2-Ethyl-5-methyl-pyrazine	13360-64-0	1393	1376
8	C_7_H_10_N_2_	2-(n-propyl)-Pyrazine	18138-03-9	1397	1404
9	C_6_H_6_N_2_	Ethenyl-pyrazine	4177-16-6	1393	1429
10	C_8_H_12_N_2_	2,6-Diethyl-pyrazine	13067-27-1	1392	1410
11	C_7_H_8_N_2_	Isopropenyl-pyrazine	34413-32-6	1550	- ^d^
12	C_7_H_8_N_2_	(1-methylethenyl)-Pyrazine	38713-41-6	1597	-
13	C_7_H_8_N_2_O	1-(5-methyl-2-pyrazinyl)-1-Ethanone	22047-27-4	1684	1679
14	C_6_H_8_N_2_O	3-Methyl-2-pyrazinylmethanol	160818-32-6	1908	-
15	C_6_H_8_N_2_O	6-Methyl-2-pyrazinylmethanol	77164-93-3	2067	-
16	C_5_H_7_N	3-Methyl-1H-pyrrole	616-43-3	1571	1569
17	C_6_H_9_N	2,3-Dimethyl-1H-pyrrole	600-28-2	1601	1620
18	C_7_H_8_N_2_	1,5-Dimethyl-2-pyrrolecarbonitrile	56341-36-7	1646	1621
19	C_6_H_7_NO	1-(1H-pyrrol-2-yl)-Ethanone	1072-83-9	1970	1950
20	C_5_H_5_NO	1H-Pyrrole-2-carboxaldehyde	1003-29-8	2024	-
21	C_6_H_7_NO_2_	Methyl pyrrole-2-carboxylate	1193-62-0	2051	2058
22	C_5_H_5_N	Pyridine	110-86-1	1183	1173
23	C_6_H_7_N	2-Methyl-pyridine	109-06-8	1217	1211
24	C_7_H_10_N_2_	2,6-Dimethyl-4-pyridinamine	3512-80-9	1399	-
25	C_6_H_7_NO	3-Methoxy-pyridine	7295-76-3	1582	1579
26	C_7_H_7_NO	1-(2-pyridinyl)-Ethanone	1122-62-9	1604	1590
27	C_6_H_4_N_2_	2-Pyridinecarbonitrile	100-70-9	1901	-
28	C_5_H_5_NO	4(1H)-Pyridone	108-96-3	2414	-
29	C_8_H_8_N_2_	5-Methyl-benzimidazole	614-97-1	2093	-
30	C_8_H_7_N	Indole	120-72-9	2454	2441
31	C_4_H_5_NS	2-Methyl-thiazole	3581-87-1	1239	1239
32	C_4_H_5_NS	4-Methyl-thiazole	693-95-8	1281	1265
33	C_5_H_7_NS	2,4-Dimethyl-thiazole	541-58-2	1285	1268
34	C_5_H_7_NS	2,5-Dimethyl-thiazole	4175-66-0	1319	1326
35	C_6_H_9_NS	2-Ethyl-4-methylthiazole	15679-12-6	1345	1349
36	C_6_H_9_NOS	4-Methyl-5-thiazoleethanol	137-00-8	2306	2309
37	C_6_H_9_NS	2,4,5-Trimethyl-thiazole	13623-11-5	1379	1378
38	C_9_H_14_O	2-Pentyl-furan	3777-69-3	1230	1230
39	C_6_H_6_O_2_	5-Methyl-2-furancarboxaldehyde	620-02-0	1575	1570
40	C_7_H_8_O_2_	2-Acetyl-5-methylfuran	1193-79-9	1616	1609
41	C_7_H_8_O_2_	2-Methoxy-phenol	90-05-1	1858	-
42	C_6_H_6_O	Phenol	108-95-2	2000	1992
43	C_7_H_8_O	p-Cresol	106-44-5	2080	2091
44	C_9_H_10_O_2_	2-Methoxy-4-vinylphenol	7786-61-0	2193	2194
45	C_8_H_10_O_3_	2,6-Dimethoxy-phenol	91-10-1	2264	-
46	C_7_H_6_O_3_	1,3-Benzodioxol-5-ol	533-31-3	2681	-
47	C_6_H_14_O	3-Hexanol	623-37-0	1196	1198
48	C_8_H_10_O	Phenylethyl alcohol	60-12-8	1908	-
49	C_8_H_8_O	Acetophenone	98-86-2	1653	-
50	C_9_H_10_O_3_	1-(3-hydroxy-4-methoxyphenyl)-Ethanone	6100-74-9	2656	-
51	C_7_H_6_O	Benzaldehyde	100-52-7	1527	1502
52	C_7_H_8_	Toluene	108-88-3	1116	1075
53	C_8_H_8_	Styrene	100-42-5	1260	-
54	C_10_H_14_	n-Butyl-benzene	104-51-8	1316	1310
55	C_10_H_16_	β-Ocimene	13877-91-3	1254	1167
56	C_9_H_9_N	Benzenepropanenitrile	645-59-0	2045	2048

^a^ Volatile compounds detected in sesame oil samples obtained under different roasting conditions. ^b^ Retention index calculated with WAXETR as chromatographic column. ^c^ Retention index obtained from the NIST standard reference database (https://webbook.nist.gov/chemistry/; accessed on 18 March 2025). ^d^ “-“, not retrieved.

**Table A2:** Odor thresholds and OAVs of the compounds in sesame oil samples.

Names	OT ^a^ (mg/kg)	OAVs ^b^																	
		160-10	170-10	180-10	190-10	200-10	210-10	160-20	170-20	180-20	190-20	200-20	210-20	160-30	170-30	180-30	190-30	200-30	210-30
Methyl-pyrazine	0.06	0	0	13	33	38	26	0	0	0	0	0	0	0	0	0	0	0	381
2,5-Dimethyl-pyrazine	2.6	0	0	1	2	2	2	20	16	19	18	13	21	15	11	17	12	14	4
2,3-Dimethyl-pyrazine	0.4	0	0	0	0	0	0	3	2	4	3	2	3	3	2	4	2	3	1
2-Ethyl-6-methyl-pyrazine	0.04	0	0	0	0	0	6	269	234	308	250	201	289	282	232	367	236	256	128
2-Ethyl-5-methyl-pyrazine	0.32	0	0	0	3	3	3	28	24	29	27	22	30	27	22	34	24	25	14
2-(n-propyl)-Pyrazine	0.3	0	0	0	0	0	0	1	1	2	1	1	1	2	2	3	2	1	1
Ethenyl-pyrazine	0.7	0	0	0	0	0	0	11	9	17	10	7	18	13	8	12	8	11	3
2,6-Diethyl-pyrazine	0.006	14	5	11	29	30	24	319	268	253	322	262	338	301	249	374	256	284	160
1-(5-methyl-2-pyrazinyl)-1-Ethanone	0.4	0	0	0	0	0	0	2	1	3	2	1	3	0	2	0	2	2	1
Pyridine	2	0	0	0	0	0	0	1	0	3	2	0	1	1	1	1	0	1	0
2-Methyl-pyridine	0.01	0	0	0	0	0	0	56	0	81	54	27	60	51	0	59	0	0	0
1-(2-pyridinyl)-Ethanone	0.019	0	0	0	0	0	0	43	42	82	59	35	66	66	48	69	48	66	26
Indole	0.14	0	0	0	0	0	0	0	0	53	20	8	0	40	14	33	0	20	11
4-Methyl-thiazole	0.055	0	0	0	0	0	0	134	103	130	101	62	142	97	67	109	73	74	15
2,4-Dimethyl-thiazole	0.018	0	0	0	0	0	0	505	452	383	417	251	418	347	326	440	311	255	91
2,4,5-Trimethyl-thiazole	0.045	0	0	0	0	0	0	13	10	19	10	9	13	16	14	22	10	15	6
2-Pentyl-furan	0.006	0	0	0	0	0	0	0	0	0	0	0	0	249	132	271	124	0	0
5-Methyl-2-furancarboxaldehyde	1.11	0	0	0	0	0	0	32	24	0	29	20	55	0	0	0	0	0	0
2-Methoxy-phenol	0.02	0	0	0	0	0	0	939	815	2489	1248	701	1920	2117	1249	2157	1104	1463	755
Phenol	0.1	0	0	0	0	0	0	21	16	72	31	13	47	41	25	40	14	24	14
p-Cresol	0.025	0	0	0	0	0	0	0	0	40	10	7	15	20	0	0	0	0	0
2-Methoxy-4-vinylphenol	0.2	0	1	1	2	1	0	27	22	43	34	30	115	65	37	50	58	85	45
2,6-Dimethoxy-phenol	0.05	0	0	0	0	0	0	10	0	27	11	10	40	32	15	24	25	27	13
3-Hexanol	0.82	13	6	14	17	13	0	0	0	0	0	0	0	0	0	0	0	0	0
Phenylethyl alcohol	0.56	1	1	2	2	1	0	0	0	0	0	0	0	0	0	0	0	0	0
Acetophenone	5.629	0	0	0	0	0	0	1	1	1	1	1	1	1	1	1	1	1	0
Benzaldehyde	0.35	0	0	0	3	2	2	9	8	21	14	8	18	21	15	23	16	15	9
Styrene	0.065	2	0	1	1	0	0	0	0	0	0	0	0	0	0	0	0	0	0
n-Butyl-benzene	0.1	0	0	0	0	0	0	0	0	0	0	0	0	11	5	19	3	4	1
Benzenepropanenitrile	0.015	0	0	0	0	0	0	0	0	103	0	0	0	57	0	91	0	0	0

^a^ OT was referenced from the book *Odor Thresholds: Compilations of Odor Threshold Values in Air, Water and Other Media* (second enlarged and revised edition) [47]. ^b^ OAVs refer to the odor activity values.
